# A multi-division convolutional neural network-based plant identification system

**DOI:** 10.7717/peerj-cs.572

**Published:** 2021-05-28

**Authors:** Muammer Turkoglu, Muzaffer Aslan, Ali Arı, Zeynep Mine Alçin, Davut Hanbay

**Affiliations:** 1 Faculty of Engineering, Department of Software Engineering, Samsun University, Samsun, Turkey; 2 Engineering Faculty, Electrical and Electronics Engineering Department, Bingol University, Bingöl, Turkey; 3 Engineering Faculty, Computer Engineering Department, Inonu University, Malatya, Turkey; 4 Vedat Topçuoğlu Anatolian Vocational High School, Electrical and Electronics Department, Gaziantep, Turkey

**Keywords:** Plant Identification System, Deep features, Support Vector Machine, Principal component analysis, Division process

## Abstract

**Background:**

Plants have an important place in the life of all living things. Today, there is a risk of extinction for many plant species due to climate change and its environmental impact. Therefore, researchers have conducted various studies with the aim of protecting the diversity of the planet’s plant life. Generally, research in this area is aimed at determining plant species and diseases, with works predominantly based on plant images. Advances in deep learning techniques have provided very successful results in this field, and have become widely used in research studies to identify plant species.

**Methods:**

In this paper, a Multi-Division Convolutional Neural Network (MD-CNN)-based plant recognition system was developed in order to address an agricultural problem related to the classification of plant species. In the proposed system, we divide plant images into equal nxn-sized pieces, and then deep features are extracted for each piece using a Convolutional Neural Network (CNN). For each part of the obtained deep features, effective features are selected using the Principal Component Analysis (PCA) algorithm. Finally, the obtained effective features are combined and classification conducted using the Support Vector Machine (SVM) method.

**Results:**

In order to test the performance of the proposed deep-based system, eight different plant datasets were used: Flavia, Swedish, ICL, Foliage, Folio, Flower17, Flower102, and LeafSnap. According to the results of these experimental studies, 100% accuracy scores were achieved for the Flavia, Swedish, and Folio datasets, whilst the ICL, Foliage, Flower17, Flower102, and LeafSnap datasets achieved results of 99.77%, 99.93%, 97.87%, 98.03%, and 94.38%, respectively.

## Introduction

Plants are vital to life in general both for the environment and for humankind, and the global ecology cannot exist without plant life. Humans extensively use plants in many fields, including energy, industry, food, and also in medicine (Türkoğlu & Hanbay, 2015; Nijalingappa & Madhumathi, 2016; Bertrand et al., 2018). According to the literature, there are approximately 500,000 known plant species worldwide. As a result of the research conducted by experts in the field, new plant species are still being identified and therefore the number of existing plant species is still increasing. However, at the same time generations of certain plant species are at risk of extinction due to adverse seasonal conditions and from the detrimental effects of environmental pollution. Therefore, studies related to the protection of plants and for plant recognition are of significant importance for the determination of new plant species and the protection of existing plant life (Türkoğlu & Hanbay, 2015; Nijalingappa & Madhumathi, 2016; Türkoğlu et al., 2016, Bertrand et al., 2018) and (López-Jiménez et al., 2019).

Today, the classification of plant species is largely conducted according to traditional methods. However, it is known that such processes involve certain difficulties as they are both time-consuming and complex processes. In conjunction with advances in computer technology, many studies have been undertaken in the area of object recognition. In this context, data previously examined by individuals can now be made easier, error-free and automated without wasting time unnecessarily thanks to computer-based image processing techniques (Türkoğlu & Hanbay, 2015; Nijalingappa & Madhumathi, 2016; Türkoğlu et al., 2016; Türkoğlu & Hanbay, 2019a).

Plant classification presents a difficult problem area due to the diversity of plant species and the similarities between plant families (Türkoğlu et al., 2016) In recent years, numerous studies based on machine learning and image processing algorithms have been conducted to address the problem of plant recognition. In most studies, traditional methods based on shape, color and texture properties have been used, although more recent studies on the recognition of plant species have been conducted using deep learning methods. A review of these previous studies is detailed in ‘Related works’.

In the current study, we developed a Multi-Division Convolutional Neural Network (MD-CNN)-based plant recognition system for the classification of plant species. In this study, plant images were equally divided into *n* × *n*-sized pieces using the dividing approach, and then deep features were extracted for each piece. Effective features were chosen from the deep features obtained using the PCA method. Finally, these features obtained from the split parts were combined and classified and tested using the SVM method. In the experimental studies, eight different plant datasets were used to test the performance of the proposed MD-CNN model. According to the results of the experimental studies, it was determined that the MD-CNN model provides a superior performance to the state-of-the-art methods.

The contributions of the proposed MD-CNN model developed based on the division approach for the classification of plant species are as follows:

• In this study, plant images were divided into nxn-sized pieces using the dividing approach. Then, a CNN-based model was used to extract deep features from the split parts, and distinguishing and effective features were then selected using the TBA method. According to the results of the experimental studies, this proposed model offers a superior performance over the existing studies performed to date. As a result, this approach is considered to be an important factor in enhancing the performance of future methods developed for the solution of pattern recognition problems.
• Using the division approach, the individual performances of pretrained deep architectures were increased. In this context, it was proven that the division approach can be used as an alternative method to improve classification performance rather than increasing the number of layers.
• The proposed model is simple to implement and is not reliant upon any complex mathematical background.
• The proposed MD-CNN model was tested using eight different datasets commonly used in the literature. As a result, accuracy scores were calculated for the Flavia, Swedish, ICL, Foliage, Folio, Flower17, Flower102, and LeafSnap datasets as 100%, 100%, 99.77%, 99.93%, 100%, 97.87%, 98.03%, and 97.8%, respectively. The proposed model was compared to previous studies, and was observed to be more successful against all datasets.

The remainder of this paper is organized as follows. ‘Related works’ introduces the published literature on plant recognition methods, whilst ‘Material and methods’ presents the materials and methods of the study, including its theoretical background and the datasets used. The proposed model is detailed in ‘Proposed model’, and the experimental works and results are presented in ‘Experimental studies’. Finally, the results of the study are discussed in ‘Conclusion’.

### Related works

Recently, many studies based on machine learning algorithms have been conducted for the recognition of the plant species. In most of these studies, leaf-based plant species such as Flavia (Wu et al., 2007), Swedish (Söderkvist, 2001), ICL (Silva, Marcal & Da Silva, 2013) Foliage (Kadir et al., 2011), Folio (Munisami et al., 2015) and LeafSnap (Barré et al., 2017), as well as Flower17 (Nilsback & Zisserman, 2007; Nilsback & Zisserman, 2006) and Flower102 (Nilsback & Zisserman, 2008) real-time datasets have been widely used. The academic studies based on shape, texture and color features using these datasets are summarized in Table 1.

**Table 1:** Plant recognition studies based on traditional methods.

Researchers	Feature extraction methods	Classification methods	Datasets	Accuracy score (%)
Wu et al. (2007)	Shape features	Probabilistic Neural Network (PNN)	Flavia	90.00
Silva, Marcal & Silva (2013)	Shape features	Linear Discriminant Analysis (LDA)	ICL	87.00
Naresh & Nagendraswamy (2016)	Improved Local Binary Pattern (LBP)	k-Nearest Neighbors (k-NN)	Flavia	97.55
			Swedish	96.83
			Foliage	90.62
Tsolakidis, Kosmopoulos & Papadourakis (2014)	Zernike Moment & histogram of oriented gradients (HOG)	Support Vector Machine (SVM)	Flavia	97.18
			Swedish	98.13
Kadir et al. (2012)	Shape, color, texture features & PCA	PNN	Flavia	95.00
			Foliage	93.75
Elhariri, El-Bendary & Hassanien (2014))	Shape, color & texture features	LDA	ICL	92.65
Wang, Liang & Guo (2014)	Dual-scale decomposition & local binary descriptors	k-NN	Flavia	99.25
			ICL	98.03
Saleem et al. (2019)	Shape & texture features	k-NN	Flavia	98.75
Munisami et al. (2015)	Shape & color features	k-NN	Folio	87.30
Ren, Wang & Zhao (2012)	Multi-scale overlapped block LBP	SVM	Swedish	96.67
Sulc & Matas (2015)	Rotation & scale invariant descriptor based on LBP	SVM	Flavia	99.50
			Foliage	99.00
			Swedish	99.80
			Folio	99.20
Qi et al. (2014)	Pairwise Rotation Invariant Co-occurrence Local Binary Pattern	SVM	Swedish	99.38
			Flower102	84.20
Hewitt & Mahmoud (2018)	Shape features & signal features extracted from local area integral invariants (LAIIs)	SVM	Flavia	96.60
			Foliage	93.10
			Folio	91.40
			Swedish	97.80
			LeafSnap	64.90
Zhu et al. (2015)	Shape & color features	SVM	Flower17	91.90
			Flower102	73.10

In Table 1, the methods and datasets used in studies related to plant recognition and performance results are presented. Overall, these studies were generally conducted using shape-based methods. According to the results obtained from each study’s experimental works, the highest performance for the Flavia, Foliage, Swedish and Folio datasets was obtained by Sulc & Matas (2015), Šulc & Matas (2017), whereas the highest performance for the real-time datasets Flower17 and Flower102 was calculated as 91.9% (Zhu et al., 2015) and 84.2% (Qi et al., 2014) respectively.

Most of the previous studies undertaken for the recognition of plant species were conducted using shape, texture, and color features. The major disadvantage of these methods is that they require a preprocessing stage and are therefore unsuited for application against real-time systems. Recently, the application of CNNs (Convolutional Neural Networks) have addressed the problems caused by classical learning, and classification performance has been greatly improved as a result. Previous studies that have used deep learning for the classification of plant species are summarized in Table 2.

**Table 2:** Plant recognition studies based on deep learning.

Researchers	Feature extraction methods	Classification methods	Datasets	Accuracy score (%)
Beikmohammadi & Faez (2018)	MobileNet architecture	Logistic regression classifier	Flavia	99.60
			LeafSnap	90.54
Cıbuk et al. (2019)	AlexNet & VGG16 architectures	SVM	Flower17	96.39
			Flower102	95.70
Pawara et al. (2017)	AlexNet & GoogLeNet architectures	CNN (Fine-tuning)	Swedish	99.92
			Folio	98.60
Zhang et al. (2015)	7-layer CNN architecture		Flavia	94.60
Wick & Puppe (2017)	9-layer CNN architecture	Flavia	99.81
			Foliage	99.40
Barré et al. (2017b)	17-layer CNN architecture	Flavia	97.90
			Foliage	95.60
			LeafSnap	86.30
Lee et al. (2017)	AlexNet architecture	Multilayer Perceptron Classifier (MLP)	Flavia	99.50
			Folio	99.40
Sulc & Matas (2015)	ResNet152 & Inception-ResNetv2 architectures based on LBP	CNN (Fine-tuning)	Flavia	99.80
			Foliage	99.30
			Swedish	99.80
			LeafSnap	83.70
Kaya et al. (2019)	AlexNet & VGG16 architectures	CNN (Fine-tuning) & LDA	Flavia	99.10
			Swedish	99.11
Xiao et al. (2018)	Inceptionv3 with Attention Cropping		Flower102	95.10

Table 2 shows the datasets and performance results used in studies based on deep learning for plant recognition. According to the results obtained from deep learning-based studies, approximately a 99% performance level was established for the Flavia, Foliage, Swedish, and Folio datasets. On the other hand, for the Flower17 and Flower102 datasets, (Cıbuk et al., 2019) achieved highest accuracy scores of 96.39% and 95.70%, respectively. As a result, studies based on deep learning have provided superior performance over traditional methods.

## Material and Methods

The theoretical background of the methods used for the proposed model are detailed in the following subsections.

### Deep architectures

Recently, many deep learning architectures have been developed using Deep Evolutionary Neural Networks and large datasets. These architectures were trained using a subset of the ImageNet dataset in the ILSVRC competition. This dataset contains more than one million images, including 1,000 classes such as keyboard, mouse, pen and many animals. The current study used the ResNet101 and DenseNet201 architectures, which are both pretrained CNN models. The characteristics of these architectures are presented in Table 3.

**Table 3:** Characteristics of deep architectures.

Model	Depth	Size (MB)	Parameters (millions)	Image Input Size
ResNet101 (He et al., 2016)	101	167	44.6	224 × 224
DenseNet201 (Huang et al., 2017)	201	77	20.0	224 × 224

**Figure 1:**
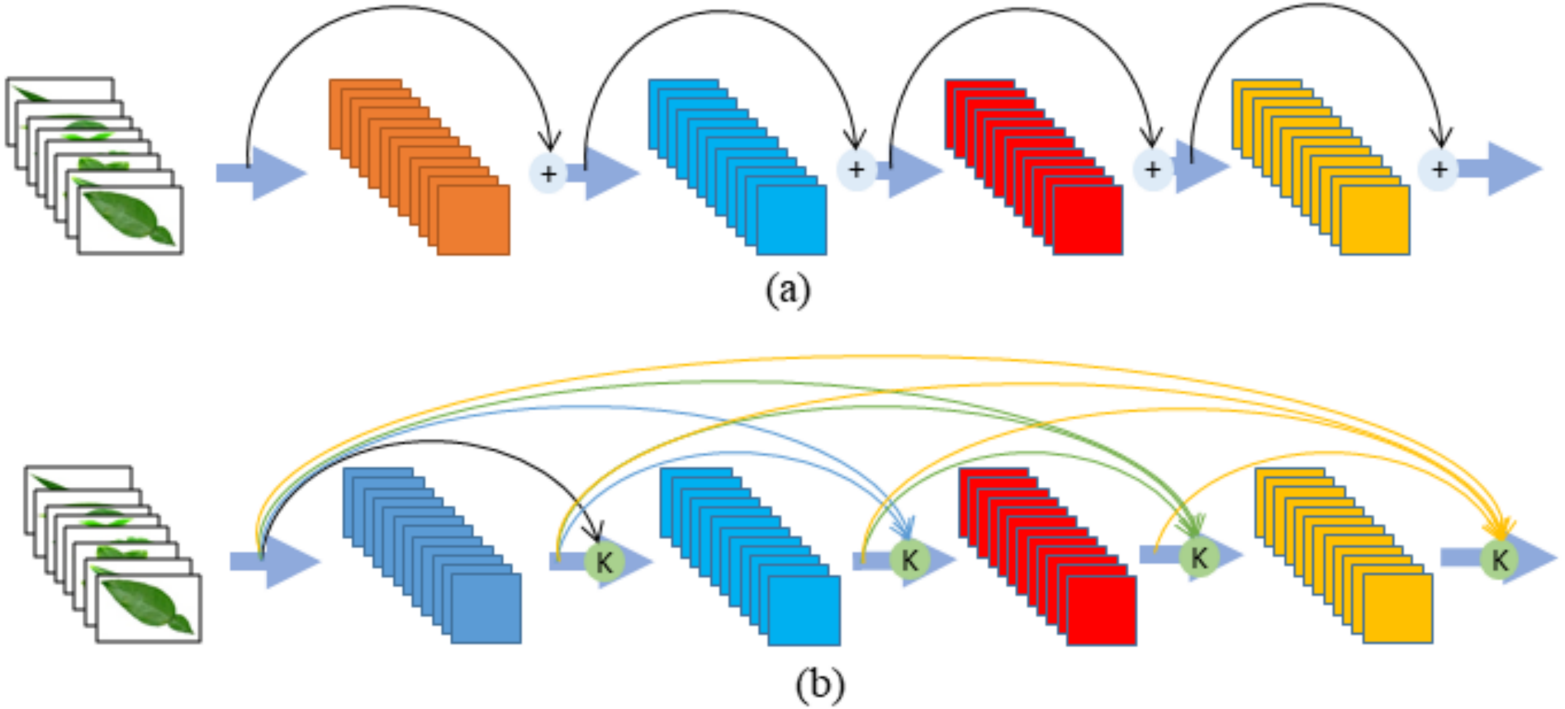
(A) ResNet (B) DenseNet Module.

The ResNet architecture (He et al., 2016) is different from sequential traditional network architectures such as VGGNet and AlexNet. Although this architecture is much deeper than the VGGNet network, the size of the network and the number of parameters are lower. The ResNet architecture is based on adding blocks that feed the next layers of values into the model (see Fig. 1A). The ResNet architecture with this structure has ceased to be a classic model and this architecture contains fewer parameters, although the depth increases (Doğan & Türkoğlu, 2018; Türkoğlu & Hanbay, 2019b).

The DenseNet and ResNet models have similar architectures. However, whilst each ResNet module in a ResNet architecture only receives information from the previous module, the DenseNet architecture is based on receiving information from the previous layers. This difference in DenseNet architecture intensively combines each layer with feedforward (Türkoğlu & Hanbay, 2019b; Nguyen et al., 2018; Chen et al., 2017; Rafi et al., 2018; Liang et al., 2018). The advantages of the DenseNet architecture over ResNet are as follows (Tsang, 2018):

• Provides strong gradient flow;
• Increases efficiency in parameter and calculation;
• More diversified features are obtained.

### Principal component analysis

Principal Component Analysis (PCA) is a method that was proposed by Hotelling in 1933. The method refers to the multivariate analysis used in basic spectral decomposition of a coefficient of coordination or covariance matrix. PCA is a statistical method that is widely used in areas such as image processing, speech recognition, text processing, pattern recognition, and image compression. Due to the complexity of the accounts in this method, computer usage was not preferred until it became widespread Hernandez, Alferdo & Türkmen, 2018. The method analyses data defined by several dependent variables associated with each of the observations (Moore, 1981). PCA finds a pattern among the variables in the original data, reducing the size of the data without loss in most of the data (Jollife & Cadima, 2016). In the PCA approach, when the variance of the original variables in the data is at the maximum, it creates a new series of orthogonal axes at an appropriate angle (Jollife & Cadima, 2016; Hernandez, Alferdo & Türkmen, 2018). The greatest change directions of the vertical axis formed in this way are called the main component. Thus, the dimensionality of the data is reduced and the interpretation of the new data becomes less complex following the conversion, as about 20 PCA components can be sufficient to represent the original data with 90% to 95% accuracy. In this regard, PCA is widely preferred for image processing applications since there is relatively little data loss while reducing data.

### Support Vector Machine

Support Vector Machine (SVM) classification is one of the most effective and simple methods used (Burges, 1998). SVMs were originally designed to classify two-class linear data. However, later it started to be used in both classification and nonlinear data classifications. Its basic principle is based on finding the best hyperplane to separate into two classes of data (Aslan et al., 2017; Demir et al., 2020). However, whilst there are many hyperplanes that can separate two-class data, SVM is able to find the hyperplane that will maximize the distance between the points closest to it.

Let us assume that SVM has training data in the form of *l* samples $ \left( {x}_{i},{y}_{i} \right) =1,2,3, \ldots , l$. Here, *x*_*i*_ shows the N-dimensional space and *y*_*i*_ ∈ { − 1, 1} class labels. Accordingly, the decision function that will find the best hyperplane, which is the purpose of the classification, can be written as shown in Eq. (1).

(1) \begin{eqnarray*}{y}_{i}({w}^{t}x+b)\geq 1\end{eqnarray*}

where *w* is the *n* dimensional weight and *b* defines the threshold value. The maximum distance $d=max( \frac{1}{ \left\| w \right\| } )$ is taken; thus, ${ \left\| w \right\| }^{2}$ is minimized and the best hyperplane is found. However, this equation only works if the predicted and actual answers have the same sign. Therefore, the product of both can only happen when there is at least one. This situation defines a major problem for SVM, but can be solved using the Lagrange optimization method of Eq. (2).

(2) \begin{eqnarray*}L \left( \alpha \right) =\sum _{i=1}^{N}{\alpha }_{i}- \frac{1}{2} \sum _{i,j=1}^{N}{\alpha }_{i}{\alpha }_{j}{y}_{i}{y}_{j}k({y}_{i},{y}_{j})\end{eqnarray*}

where, *k*(*y*_*i*_, *y*_*j*_) is the kernel function and *α*_*i*_ are the Lagrange multipliers.

### Datasets

In the current study, we used eight different datasets, namely Flavia, Swedish, ICL, Foliage, Folio, Flower17, Flower102, and LeafSnap, to test the performance of the proposed MD-CNN model. A large number of plant recognition studies have been conducted in the literature using these same datasets. The characteristic features and sample images such as the number of images, number of species, and the image sizes related to these datasets are presented in Table 4.

**Table 4:** Characteristics and sample images of datasets.

Dataset	Samples Images			Number of species	Number of species	Image dimensions
Flavia	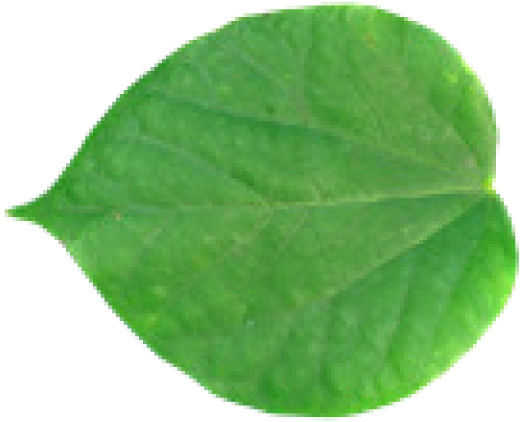	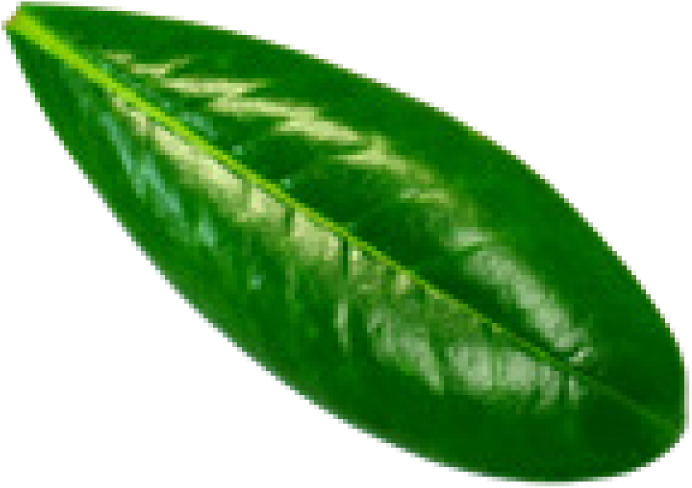	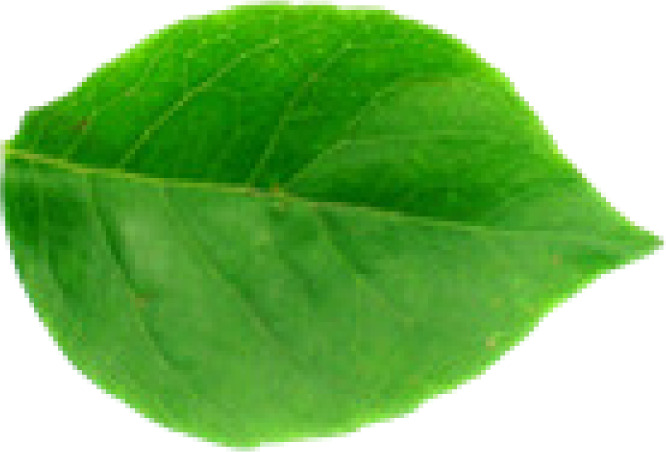	1,907	32	1,600 × 1,200
Foliage	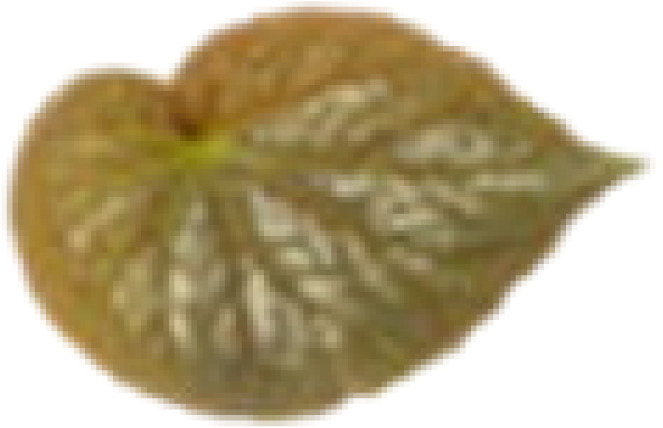	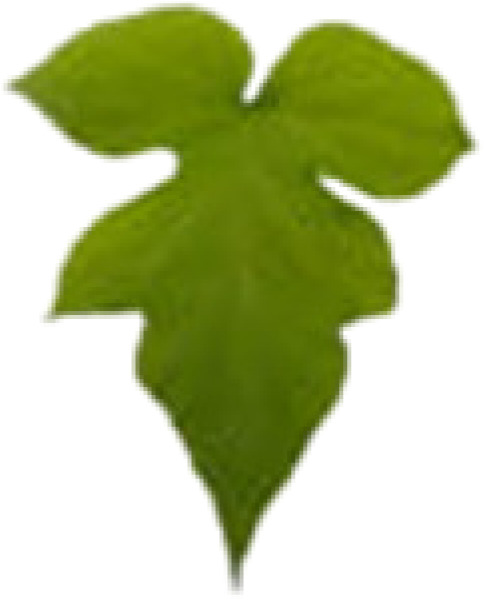	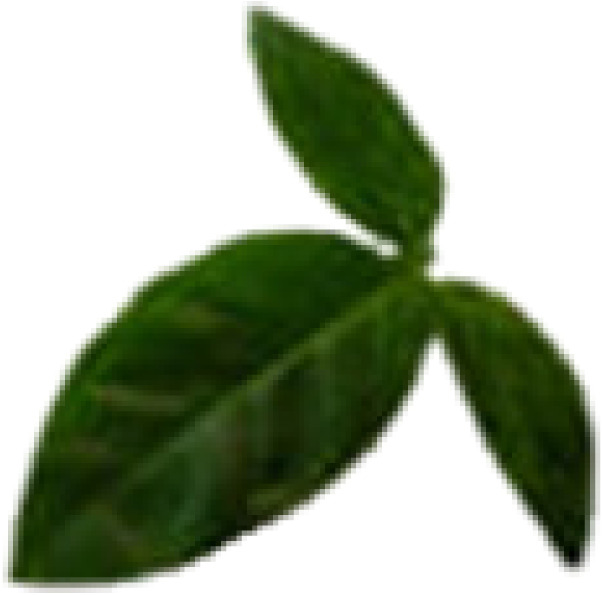	7,200	60	–
Folio	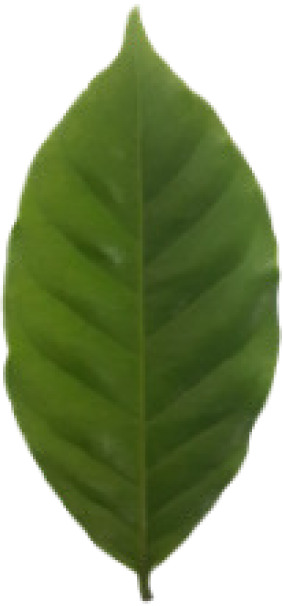	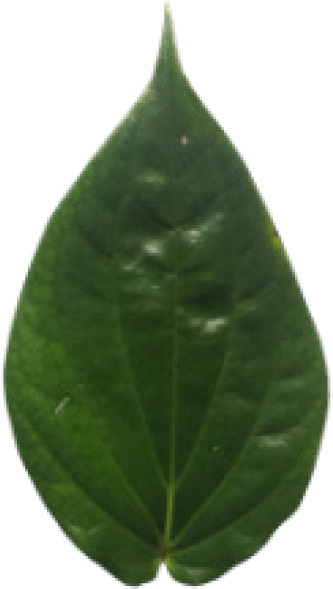	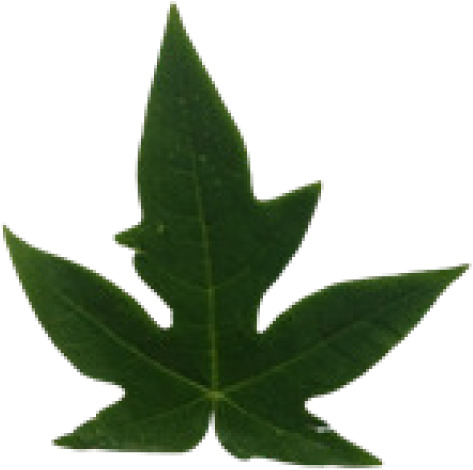	640	32	2,322 × 4,128
Swedish	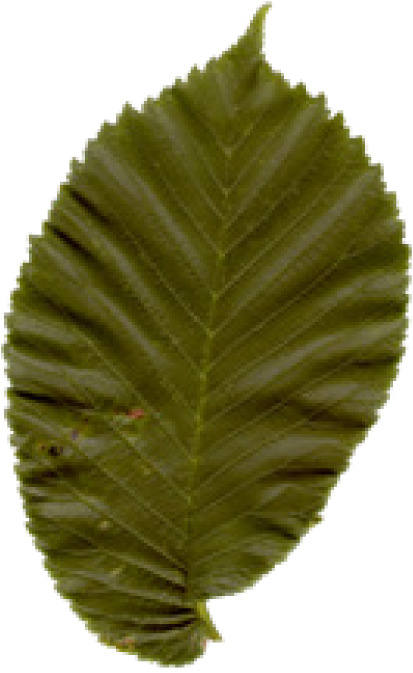	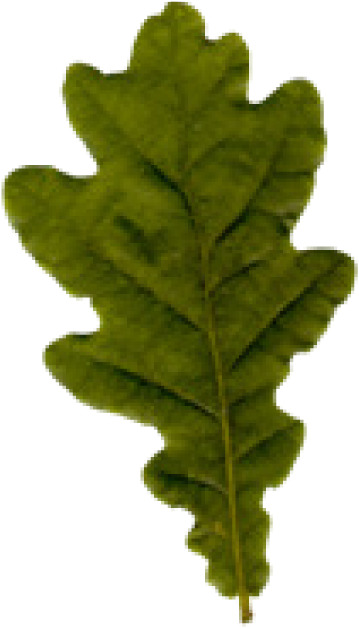	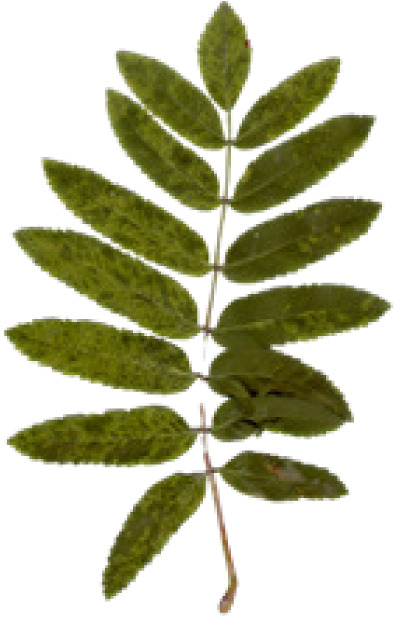	1,125	15	–
LeafSnap	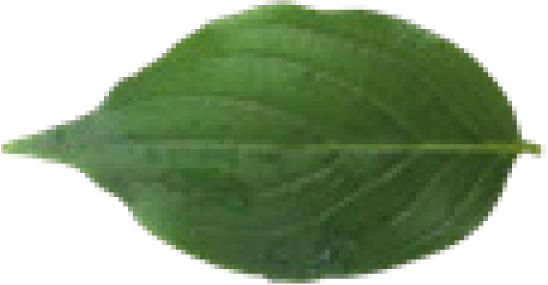	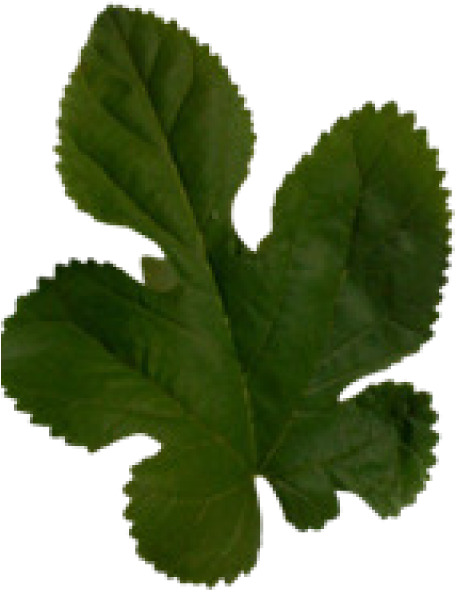	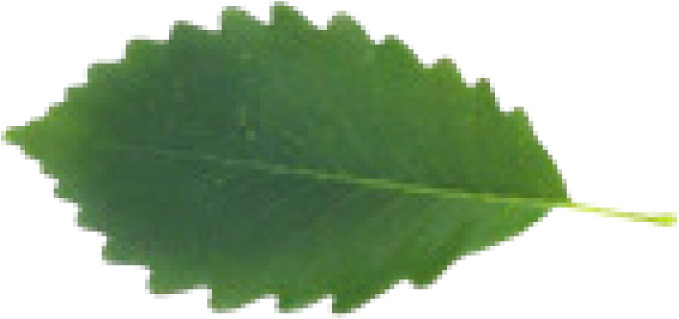	7,719	185	–
Flower17	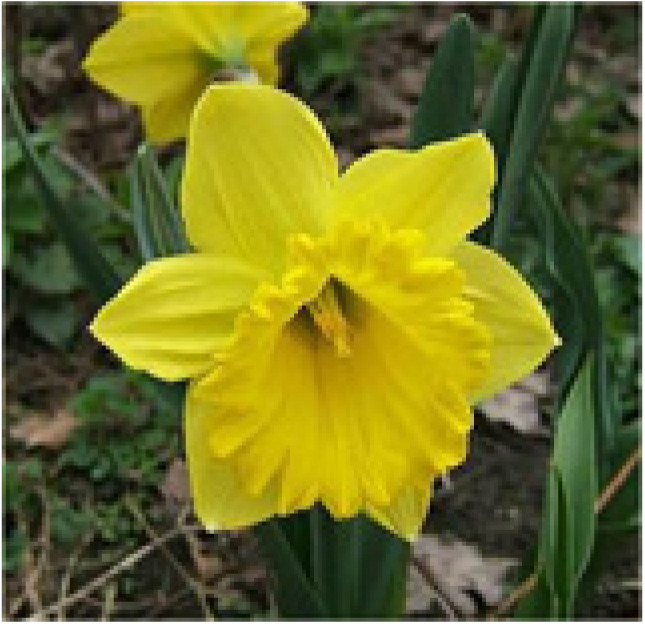	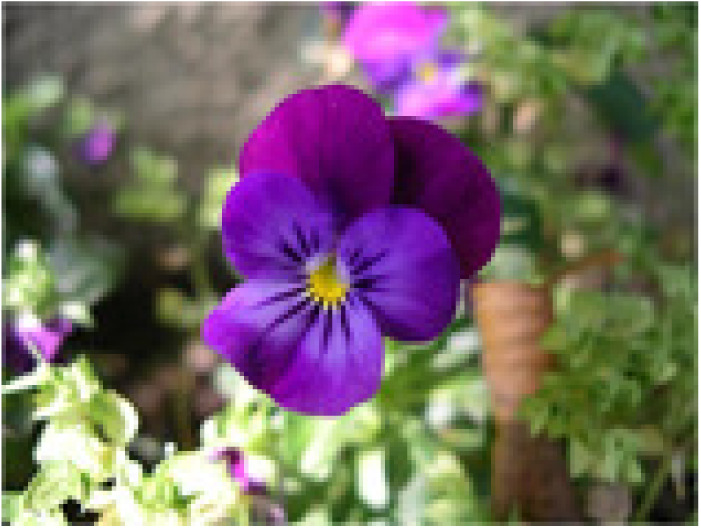	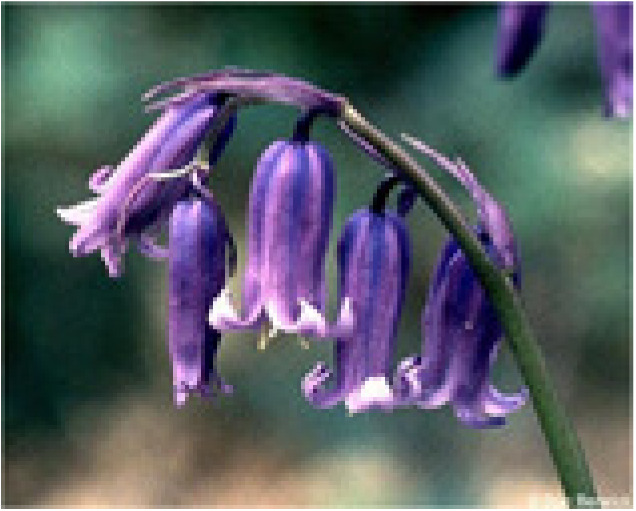	1,360	17	–
Flower102	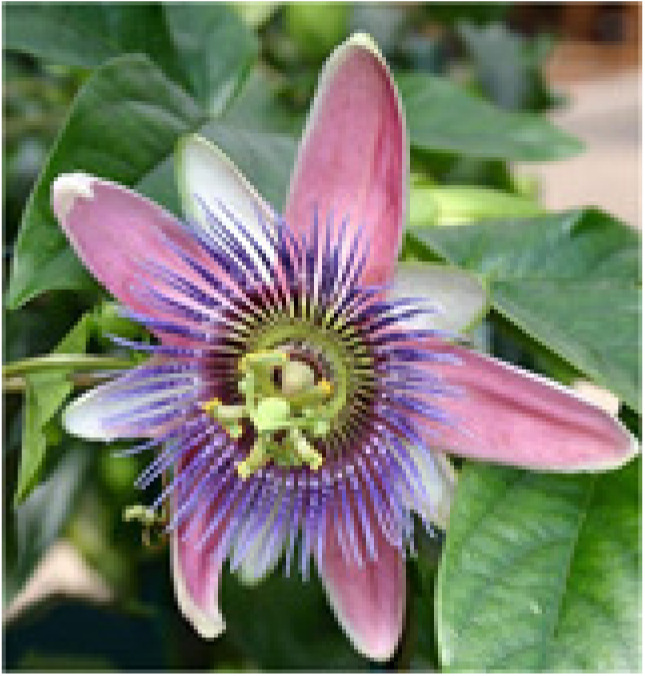	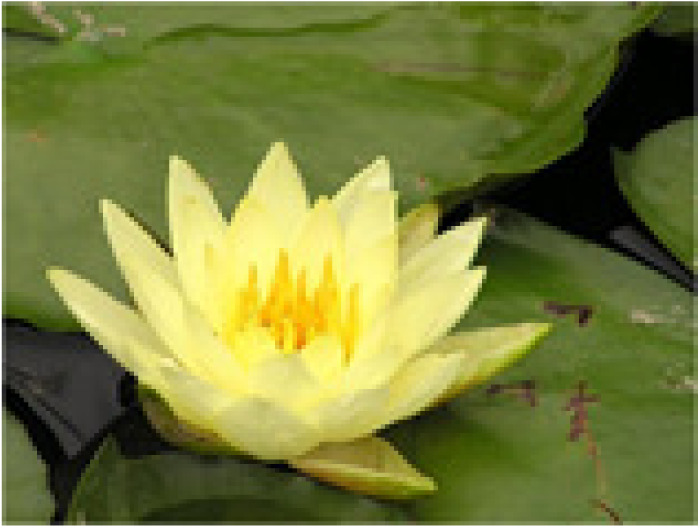	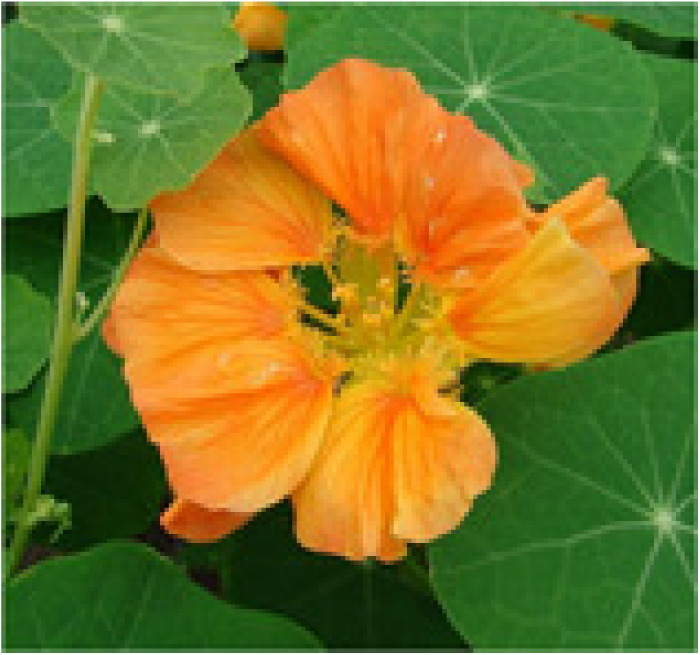	8,189	102	–

## Proposed Model

**Figure 2:**
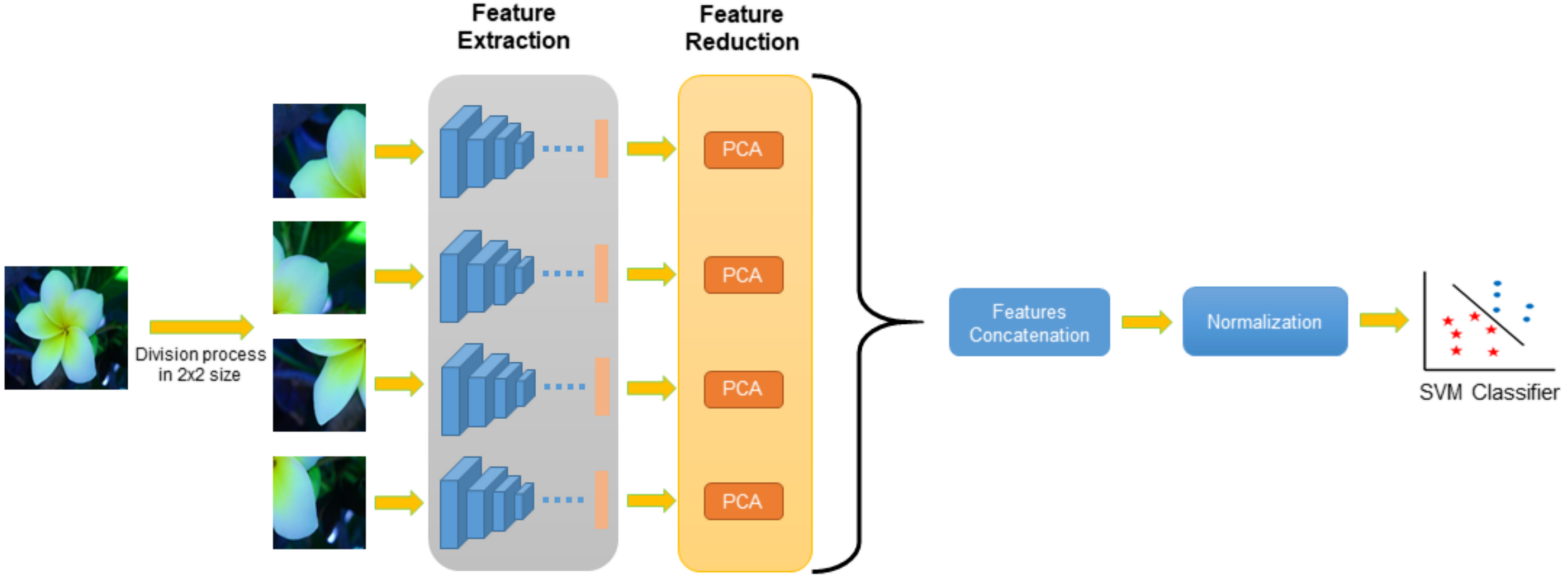
General flowchart of MD-CNN model.

In this study, a system based on deep convolutional neural networks was developed by using the division approach. A general flowchart of the proposed Multi-Division Convolutional Neural Network (MD-CNN) system is shown in Fig. 2.

Before applying the MD-CNN model, a preprocessing phase was employed for the Flavia, Folio, ICL, Swedish, Foliage, and LeafSnap datasets. The background of the images in these datasets are monochrome, red or white. Since background other than the leaf image will not contribute to the plant classification performance and no distinctive information will be obtained, cropping is applied to remove this background using the following process:

Step 1: Obtain a plant image (image).

Step 2: Convert RGB image to grayscale.

\begin{eqnarray*}R=image  \left( :,:,1 \right) \end{eqnarray*}

\begin{eqnarray*}G=image (:,:,2) \end{eqnarray*}

\begin{eqnarray*}B=image (:,:,3) \end{eqnarray*}

\begin{eqnarray*}img\text{_}gray=R~\ast ~0.2989+G~\ast ~0.5870+B~\ast ~0.1140 \end{eqnarray*}

Step 3: Apply max–min normalization.

\begin{eqnarray*}img\text{_}norm= \frac{{img}_{gray}-min({img}_{gray})}{\mathrm{max}({img}_{gray}) -min({img}_{gray})} \end{eqnarray*}

Step 4: Obtained image < *T* (*T* is the best thresholding).

Step 5: Fill image regions and holes, erode process of image and select objects in binary image.

Step 6: Obtain the boundary curves of the leaf object.

Step 7: Perform cropping process using the obtained boundary curves.

**Figure 3:**
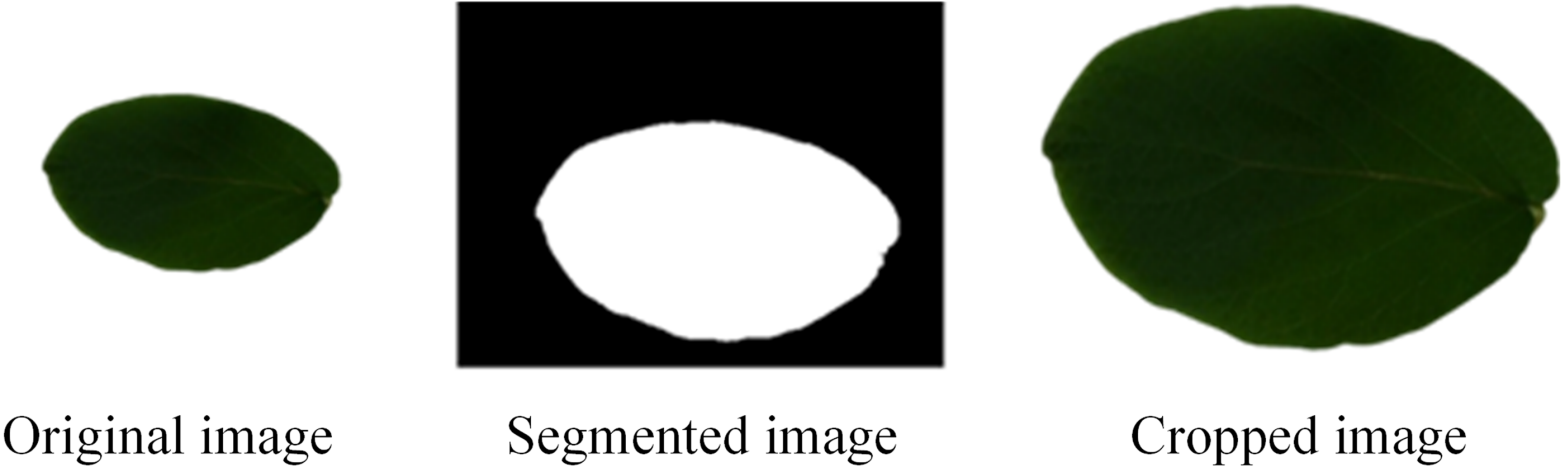
Cropping process application.

By applying these operational process steps, the leaf image is separated from the background, as shown in the example in Fig. 3.

The application steps for the general flowchart of the MD-CNN model are given in Fig. 3, and are detailed as follows:

Step 1: Obtain a plant image.

Step 2: Divide the plant image into *nxn* sized matrices.

Step 3: Resize the divided plant image according to the structure of deep nets using Bilinear interpolation.

Step 4: Extract deep features for each divided image using pretrained CNN architectures.

Step 5: Select effective features from the deep features obtained for each divided image using the PCA method.

Step 6: Combine selected features for each divided image.

Step 7: Apply normalization process.

Step 8: Apply the SVM method to classify the obtained properties

For the proposed nxn division approach, four different examples (2 × 2, 3 × 3, 4 × 4, 5 × 5) were evaluated. A total of 1,000 deep features were extracted from the divided plant images using the deep architectures, which were developed based on deep convolutional neural networks. Distinctive features were then selected for these deep features of using the PCA method. The selected features obtained for each part were combined and normalization was applied using the *z*-score method. All these processes for the DenseNet201 and ResNet101 architectures were applied separately and the features obtained were combined prior to the classification stage. Finally, the SVM method was employed in the classification phase.

**Table 5:** Performance results of proposed MD-CNN model.

Dataset	Flavia	Swedish	ICL	Foliage	Folio	Flower17	Flower102	LeafSnap
Accuracy score	100%	100%	99.77%	99.93%	100%	97.87%	98.03%	94.38%
Feature numbers	240	320	1,620	2,700	910	1,720	1,600	4,480

## Experimental Studies

In this paper, we propose a Multi-Division Convolutional Neural Network (MD-CNN) model based on the nxn-sized division approach. In addition, plant images were divided into parts using window sizes of 2 × 2, 3 × 3, 4 × 4, and 5 × 5, and then deep features were extracted using pretrained architectures for each piece. Using the PCA method, effective parameters were selected from the obtained deep features and these effective features were combined. The proposed MD-CNN model then calculated the performances by using the SVM classifier. The parameters of this classifier were determined as the 10-fold cross-validation method, Quadratic kernel function, and the one-vs-all approach.

Extensive experimental studies were conducted on eight datasets commonly used in the literature to test the performance of the proposed MD-CNN model. MATLAB software was used for all experimental studies, and the computer used in the experiments had an i7 2.8 GHz processor, GTX 950 m 8 GB GPU card and 16 GB RAM.

The following subsections detail the experimental results and performance comparisons.

### Performance results

The deep-based system proposed in the current study for the classification of plant species had accuracy scores calculated by using 2 × 2, 3 × 3, 4 × 4 and 5 × 5 matrix division processes. In the experimental studies that were conducted, the highest accuracy score was achieved by using the proposed 2 × 2 division approach for the Flavia, Swedish, Flower17, Flower102, and LeafSnap datasets; while for the ICL, Foliage, and Folio datasets, the highest accuracy score was obtained by using the 3 × 3 division approach. These results and the corresponding feature numbers are presented in Table 5.

**Figure 4:**
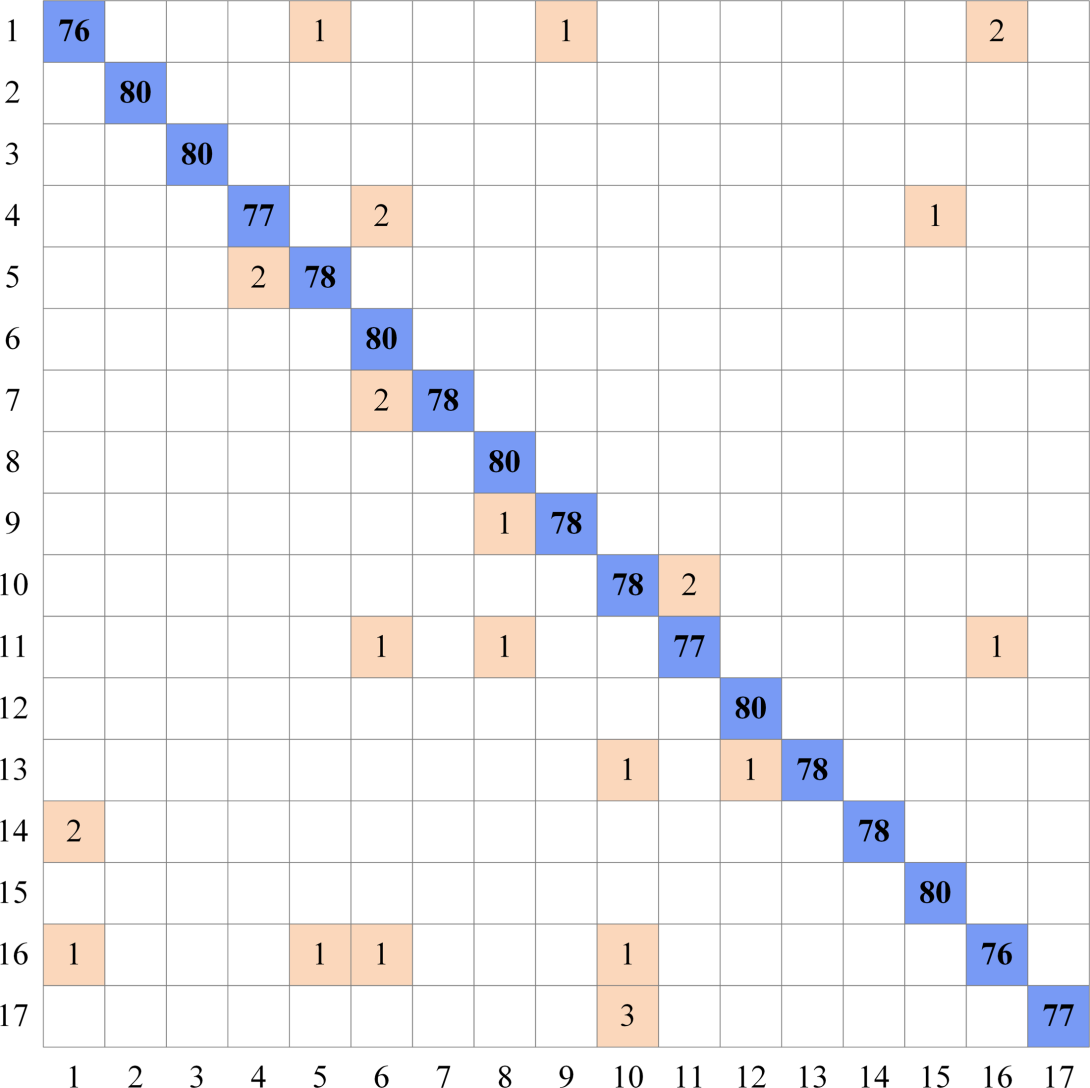
Complexity matrix for Flower17 dataset.

As can be seen in Table 5, the highest accuracy scores are shown for eight different datasets using the proposed MD-CNN model. According to these results, a 100% accuracy score was obtained for the Flavia, Swedish, and Folio datasets, while the accuracy scores obtained for the ICL, Foliage, Flower17, Flower102, and LeafSnap datasets were 99.77%, 99.93%, 97.87, 98.03%, and 94.38%, respectively. The complexity matrix of the highest accuracy score obtained for the Flower17 dataset using the MD-CNN model is shown in Fig. 4.

In Table 5, the feature parameter numbers are given using the PCA method for each dataset. For example, with ResNet101 by using the 2 × 2 division approach for the Flavia dataset, a total of 30 features were extracted from each of the four sections obtained from a raw image, making a total of 120 features having been obtained. Similarly, 120 features were obtained for DenseNet201. Then, these features were combined and applied to SVM.

Based on the division approach, extensive experimental studies were conducted for each of the eight datasets. For example, a 100% accuracy score was obtained for the Flavia dataset when using the 2 × 2, 3 × 3, 4 × 4, and 5 × 5 division approaches. In addition, an accuracy score of 99.93% was achieved when using the 3 × 3 division approach for the Foliage dataset, whilst accuracy scores of 99.9%, 99.87%, and 99.72% were obtained for the 2 × 2, 4 × 4, and 5 × 5 division approaches, respectively. Across all of the experimental studies, it was observed that the highest performances for each dataset were obtained from the 2 × 2 and 3 × 3 division approaches.

### Comparison of proposed model with other studies

The accuracy scores of the proposed MD-CNN model were compared with the state-of-the-art studies in the literature for the recognition of plant species. These comparative results are presented in Table 6 considering the datasets used in the current study.

**Table 6:** Comparison of accuracy scores from previous studies with MD-CNN model (%).

Researchers	Flavia	Swedish	ICL	Foliage	Folio	Flower17	Flower102	LeafSnap
Naresh & Nagendraswamy (2016)	97.55	96.83		90.62				
Elhariri, El-Bendary & Hassanien (2014)			92.65					
Xiao et al. (2018)							95.1	
Wang, Liang & Guo (2014)	99.25		98.03					
Yasar et al. (2015)			92.00					
Sulc & Matas (2015)	99.50	99.80		99.00	99.20			
Qi et al. (2014)		99.38					84.20	
Hewitt & Mahmoud (2018)	96.60	97.80		93.10	91.40			64.90
Khan et al. (2011)							73.30	
Zhu et al. (2015)						91.90	73.10	
Beikmohammadi & Faez (2018)	99.60							90.54
Cıbuk et al. (2019)						96.39	95.70	
Barré et al. (2017b)	97.90			95.60				86.30
Sulc & Matas (2015)	99.80	99.80		99.30				83.70
**MD-CNN model**	**100**	100	**99.77**	**99.93**	**100**	**97.87**	**98.03**	**94.38**

Table 6 shows how the experimental results of the proposed MD-CNN model compared to previous studies which had recorded high levels of performance accuracy for the eight plant image datasets used in the current study. Unlike the previous studies, the current study obtained distinctive features for each part of the image by divided the image into nxn window sizes, rather than using the whole leaf image. As can be seen in Table 6, this approach in the current study produced a significantly increased level of classification performance.

According to the results obtained in the current study’s experimental studies, the accuracy scores for the Flavia, Swedish, ICL, Foliage, Folio, Flower17, Flower102, and LeafSnap datasets were 100%, 100%, 99.77%, 99.93%, 100%, 97.87%, 98.03%, and 94.38%, respectively. Based on these results, the highest performance achieved for all eight datasets were from the proposed MD-CNN model when compared to the previous studies.

## Conclusion

In this study, a Multi-Division Convolutional Neural Network (MD-CNN) model based on nxn-sized division approach was developed for the classification of plant species. Plant images were divided into parts using window sizes of 2 × 2, 3 × 3, 4  × 4, and 5 × 5, and then deep features were extracted for each piece. Using the PCA method, effective parameters were chosen from the deep properties obtained for each part. Finally, the outstanding features obtained from the divided parts were combined and classification performance calculated using the SVM method. In the experimental studies, eight different plant datasets were used in order to test the robustness and performance of the proposed MD-CNN model. According to the results of these experimental studies, 100% accuracy scores were achieved for the Flavia, Swedish, and Folio datasets, whilst the accuracy scores for the ICL, Foliage, Flower17, Flower102, and LeafSnap datasets were 99.77%, 99.93%, 97.87%, 98.03%, and 94.38%, respectively. Performance of the proposed MD-CNN model was compared against existing studies based on the latest technology, and it was observed that the proposed model provided superior performance for all datasets. According to these results, the proposed MD-CNN model was proven to show superior performance results for use in real-world problems.

In future works, we are planning to develop models based on a combination of the Inception and ResNet residual models. In addition, various classifiers, different real-time datasets, and different feature selection methods will also be explored for the recognition of plant species.

## Additional Information and Declarations

### Data Availability

The following information was supplied regarding data availability: MATLAB codes are available at GitHub: https://github.com/mturkoglu23/Plant-Identification-System.
